# Preferences on the Timing of Initiating Advance Care Planning and Withdrawing Life-Sustaining Treatment between Terminally-Ill Cancer Patients and Their Main Family Caregivers: A Prospective Study

**DOI:** 10.3390/ijerph17217954

**Published:** 2020-10-29

**Authors:** Cheng-Pei Lin, Jen-Kuei Peng, Ping-Jen Chen, Hsien-Liang Huang, Su-Hsuan Hsu, Shao-Yi Cheng

**Affiliations:** 1 Cicely Saunders Institute of Palliative Care, Policy and Rehabilitation, King’s College London, London SE5 9PJ, UK; cp.lin@ym.edu.tw; 2 Institute of Community Health Care, School of Nursing, National Yang-Ming University, Taipei 112304, Taiwan; 3 Department of Family Medicine, College of Medicine and Hospital, National Taiwan University, Taipei 100229, Taiwan; jimmy650228@yahoo.com.tw (J.-K.P.); tennishuang@gmail.com (H.-L.H.); 4 Department of Family Medicine, Kaohsiung Medical University Hospital, Kaohsiung Medical University, Kaohsiung 80756, Taiwan; pingjen.chen@gmail.com; 5 Marie Curie Palliative Care Research Department, Division of Psychiatry, University College London, London W1T 7NF, UK; 6 Department of Family Medicine, Taipei City Hospital, Taipei 10341, Taiwan; joanna819@gmail.com

**Keywords:** advance care planning, life-sustaining treatment, medical decision-making, relational autonomy, terminal cancer

## Abstract

*Background*: The Western individualistic understanding of autonomy for advance care planning is considered not to reflect the Asian family-centered approach in medical decision-making. The study aim is to compare preferences on timing for advance care planning initiatives and life-sustaining treatment withdrawal between terminally-ill cancer patients and their family caregivers in Taiwan. *Methods*: A prospective study using questionnaire survey was conducted with both terminally-ill cancer patient and their family caregiver dyads independently in inpatient and outpatient palliative care settings in a tertiary hospital in Northern Taiwan. Self-reported questionnaire using clinical scenario of incurable lung cancer was employed. Descriptive analysis was used for data analysis. *Results*: Fifty-four patients and family dyads were recruited from 1 August 2019 to 15 January 2020. Nearly 80% of patients and caregivers agreed that advance care planning should be conducted when the patient was at a non-frail stage of disease. Patients’ frail stage of disease was considered the indicator for life-sustaining treatments withdrawal except for nutrition and fluid supplements, antibiotics or blood transfusions. Patient dyads considered that advance care planning discussions were meaningful without arousing emotional distress. *Conclusion*: Patient dyads’ preferences on the timing of initiating advance care planning and life-sustaining treatments withdrawal were found to be consistent. Taiwanese people’s medical decision-making is heavily influenced by cultural characteristics including relational autonomy and filial piety. The findings could inform the clinical practice and policy in the wider Asia–Pacific region.

## 1. Introduction

Advance care planning is a voluntary process of discussion to facilitate terminally-ill patients’ goal-concordant care when they cannot speak for themselves at some point in the future. Such discussions involve all related stakeholders (i.e., patients, family caregivers and healthcare staff) to encourage person-centered decision-making based on the patient’s values and preferences, and record the decisions in an advance directive to guide future care [1,2]. Although advance care planning is widely accepted to benefit various patients at any age and any disease stage, and their family caregivers globally [3,4,5], this Western individualistic understanding of autonomy has been debated as not reflecting the Asian social norm of using a family-centered approach in patients’ medical decision-making [6,7,8]. Adapting a culturally appropriate strategy for advance care planning delivery in a culturally acceptable context is recommended to improve its acceptability and feasibility of related decisions [6,9]. In Asia, evidence has shown that opinions from family members are highly influential and that patients made decisions by considering their relationship with and responsibility for others [10,11,12]. Such norms increase the challenges in practice not only at the individual level, but also at a wider societal level. For example, the clinical trial conducted by Tang et al. [13] in Taiwan reported that family members could override the cancer patient’s right of self-determination on medical treatments, resulting in a failure of patient’s goal-concordant care provision. A qualitative study by Menon et al. [7] elaborated public anxiety about advance care planning implementation due to different social expectations and confusion regarding the legal framework on advance care planning from the perspective of Singaporeans.

The Patient Right to Autonomy Act was adopted in Taiwan in 2019 to facilitate patients’ needs to express their preferences on certain life-sustaining treatments in advance care planning discussions. This made Taiwan the first Asian country to legalize such discussions and decisions [14]. In addition, this policy movement advocates family involvement inpatients’ advance care planning discussion to acknowledge the value of shared decision-making, which is widespread in clinical practice in Asian cultures [7,10,15]. Family members are considered to know the patients well, which means that they are often consulted by staff as proxy when crucial medical decision-making is needed. This is particularly true of advance care planning initiatives, including that the healthcare staff would request permission from family caregivers to conduct end-of-life care discussions with patients given the sensitive nature of such discussion topics. However, evidence indicates that inconsistent expectations on advance care planning between patients and family caregivers hampers the patients’ advance care planning engagement and also burdens the family surrogates [15,16,17]. This results in a delay of palliative care referrals and goal-concordant care provision as the healthcare staff has no clue about how to launch the preferred care discussions. Early advance care planning discussions are believed to improve cancer patients’ palliative care access for achieving better healthcare outcomes [18]. However, patients and family caregivers preferred timing to start an advance care planning discussion and life-sustaining treatment withdrawal are still unknown in Taiwan.

Given the evidence supporting family-led advance care planning discussions in Asia [6,10], we hypothesized that inconsistencies may be found between terminally-ill cancer patients and their family caregivers’ in the timing of initiating advance care planning and withdrawing life-sustaining treatments. Therefore, the aim of this study was to compare the preferences between terminally-ill cancer patients and their family caregivers on the timing of initiating advance care planning and withdrawing life-sustaining treatment in hospital palliative care settings in Taiwan.

## 2. Materials and Methods

### 2.1. Study Design and Theoretical Underpinning

This prospective study was conducted with terminally-ill cancer patients and their family caregivers using a questionnaire survey. This study was a part of the wider multicenter, cross-cultural advance care planning project between Taiwan and Japan, which adopted a mixed-method approach [19]. The theoretical underpinning of this study is based on the concept of ‘shared decision-making’, which emphasizes that the decision-making process for patients’ medical treatment is affected by others (e.g., family caregivers and healthcare staff). Our aforementioned hypothesis (see Introduction) and concept informed the study aim and study design, including the sampling method, data collection and data analysis.

### 2.2. Ethical Considerations

Ethical approval for the study protocol was obtained from Taiwan University Hospital Institutional Review Board (reference number: 201808114RIND). As it is a prospective study, all included patients and their family members provided signed informed consent to participate in the study.

### 2.3. Study Setting and Sampling

This study was conducted at one 17-bed inpatient hospice and palliative care unit and outpatient clinics in a single medical center in northern Taiwan. The multidisciplinary hospice and palliative care team comprising physicians, nurses, social workers, psychologists, dieticians, music therapists, chaplains and volunteers provides holistic care to more than 10,000 cancer patients with hospice and palliative care needs and their family members.

Patient participants were invited to take part in this study if they were: (1) diagnosed as having terminally-ill cancer with metastasis or recurrence and clear about their diagnosis; (2) aged 20 years old or above; (3) conscious and competent to provide informed consents; and (4) communicable in either Taiwanese or Mandarin. Family members were nominated by patients who were: (1) the primary caregiver; (2) knowing patient’s disease prognosis well; and (3) able to provide informed consents. The patients and family caregivers were matched as dyads given the aim of this study was to compare the consistency of the timing of initiating advance care planning and withdrawing life-sustaining treatments between them.

We conducted this pilot study to explore this issue empirically, so sample size calculation is not needed.

### 2.4. Recruitment and Data Collection

Guided by the research team, the physicians screened every patient in the palliative care inpatient unit and outpatient clinics for eligibility. A brief introduction of this study was provided by the physicians to the eligible patients and their family caregivers. Their contact information was then shared with the research assistant to determine if they were interested in taking part. A separate occasion was arranged for them during which the research assistant could explain the details of the study and address any relevant questions from patients and family participants. At least 24 h was offered to study participants in which to consider study participation and give their permission. The research assistant helped patients and their family caregivers to complete the questionnaire survey independently after the informed consent was obtained from both parties. An incentive equivalent to NTD 200 (approximately USD 7) was provided to both patients and family participants to appreciate for their time. A monthly ongoing meeting was conducted between research team and clinicians to discuss any obstacles regarding recruitment and data collection and resolve issues if necessary.

### 2.5. Questionnaire Development

The applied questionnaire was developed in a wider project of advance care planning, which comprised three clinical conditions of cerebral infarction, heart failure, and incurable lung cancer. The questionnaire was originally developed by Japanese academic experts and then strengthened by two Japanese specialists with expertise regarding each condition (total six). A pre-test of the questionnaire was conducted with seven patients and seven healthcare staff to ensure the clarity of the question items; this was followed by a pilot test with 23 patients to finalize the questionnaire in Japanese. For Taiwanese settings, a forward (by a bilingual physician in Taiwan) and backward (by a professional translation service) translation into Mandarin was performed afterwards [20,21]. The details of the questionnaire development have been published elsewhere [19].

In the present study, we employed only the questionnaire with the clinical condition of incurable lung cancer given the nature of participants with cancer. The questionnaire comprised three components: (1) demographic data and experience of being proxy decision makers and hospitalization; (2) a clinical scenario was provided in which to study participants and they were expected to report their preferences on the timing of initiating advance care planning based on the different frailty stages of incurable lung cancer (i.e., non-frail, early pre-frail, late pre-frail and frail).The influential factors to define frailty proposed by Fried et al. [22] were applied (e.g., weight loss; exhaustion; low physical activity, slowness and weakness) (Appendix A); and (3) their preferences on the timing of withdrawing life-sustaining treatments (i.e., intubation and resuscitation, artificial nutrition and hydration, antibiotics, blood transfusion and hemodialysis).The questionnaire for family caregivers was primarily the same as the patients’ questionnaire, with one additional question asking about the changes in family caregivers’ attitudes on advance care planning before and after they actually took care of the patients (i.e., yes, no comment or no).

### 2.6. Data Management and Analysis

Collected data were entered into Excel sheets for data management. Descriptive analysis was performed to demonstrate the sample characteristics by using frequency, percentage and range for categorical data; mean and standard deviation (mean ± SD) for continuous data. The Chi-square test or Fisher’s exact test was used to compare differences between patients and family caregivers in timing (categorical variables) of initiating advance care planning and withdrawing life-sustaining treatments. Since the dyads of patients and family caregivers are paired data, the *p* value of Chi-square test or Fisher’s exact test was not exactly correct, and it could only be considered as an approximation. All *p* values < 0.05 were regarded as statistically significant. Statistics software R version 3.6.3 (R Development Core Team, University of Auckland, New Zealand) was used for data analysis.

## 3. Results

**Figure 1:**
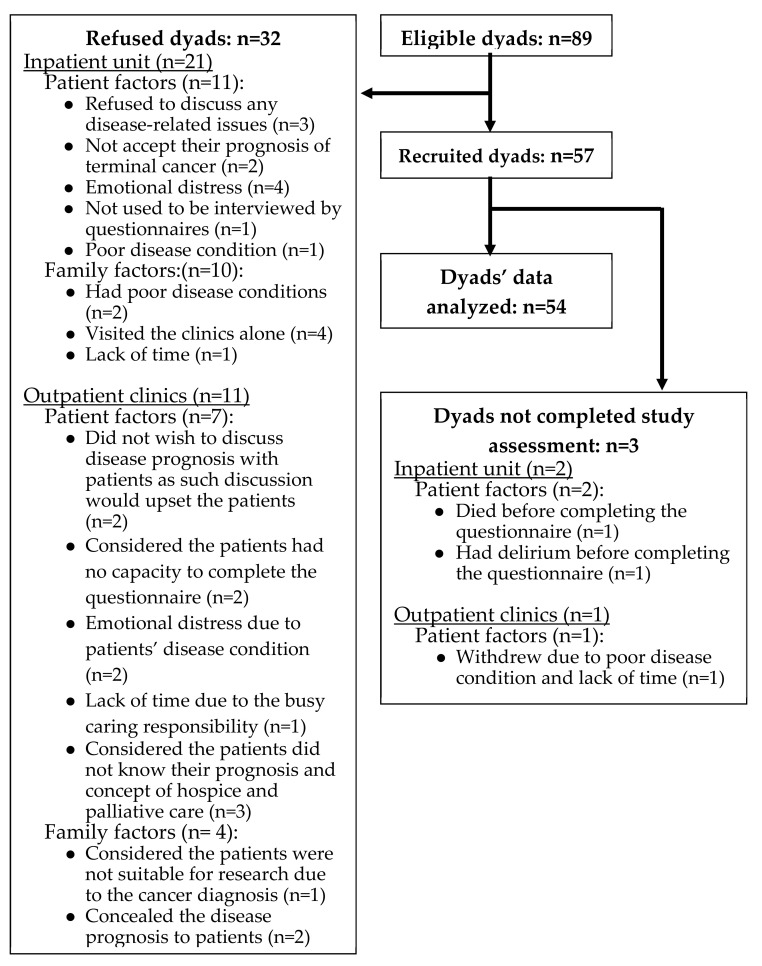
Flow diagram of study recruitment.

Fifty-four patient and family dyads were recruited between 1 August 2019 to 15 January 2020 (Figure 1). Mean age of the cancer patients was 60.64 years old (SD ± 13.90 years); mean age of family caregivers was 52.57 years old (SD ± 11.90 years). More than half of all participants were male (56% of patients and 61% of family caregivers) and married (61% of patients and 72% of family caregivers). Around half of the patients (48%) and half of the family caregivers (54%) had received higher education (university and postgraduate degrees). The family caregivers’ relationships with patients were primarily their spouses (33%), children (30%) and siblings (20%). Although more than half of the patients had hospitalization experience, the majority (69%) reported no experience in proxy decision-making. In contrast, approximately 70% of family caregivers had experience as proxy to make medical decisions for their loved ones (see Table 1).

*Preferences on the Timing of Initiating Advance Care Planning and Life-Sustaining Treatments (CPR, Intubation and Artificial Ventilator, Artificial Nutrition and Hydration, Antibiotics, Blood Transfusion and Haemodialysis) Withdrawal between Patients and Family Caregivers*

No statistical differences are found in the timing of initiating advance care planning between patients’ and family caregivers’ perspectives. Four-fifths (80%) of patients and family caregivers considered that advance care planning should be initiated when the patients are at a non-frail stage (see Table 2). The pattern for the preferred timing to withdraw life-sustaining treatment was similar between patients and family caregivers. A majority of them reported that frailty was the indicator to withdraw certain invasive life-sustaining treatments, such as cardiopulmonary resuscitation (39% of patients; 39% of family caregivers), intubation and artificial ventilation (39% of patients; 44% of family caregivers). However, artificial nutrition and hydration (33% of patients; 46% of family caregivers), antibiotics (50% of patients; 52% of family caregivers) and blood transfusion (43% of patients; 48% of family caregivers) were not to be suspended under any conditions. The decisions on haemodialysis varied and some patient (22%) and family participants (20%) felt uncertain. Most study participants (91% of patients; 94% of family caregivers) considered that it was meaningful. In addition, a majority (78% of patients; 81% of family caregivers) did not feel uncomfortable about advance care planning discussions. No difference in attitudes on advance care planning was noticed in family caregivers (74%) pre and post-patient care (see Table 3).

## 4. Discussion

This is a novel study comparing the preferred timing of advance care planning initiatives and life-sustaining treatment withdrawal for terminally-ill cancer patients from the perspectives of patient and family caregiver dyads, a situation which has received little attention in Taiwan previously. Results suggest that the tendency toward such decisions is consistent between patients and family caregivers, which does not fully support our hypothesis: ‘inconsistencies may be found between terminally-ill cancer patients and their family caregivers’ in the timing of initiating advance care planning and withdrawing life-sustaining treatments.’ Both patients and their family caregivers considered that it was appropriate to initiate advance care planning discussion earlier when the patient was at a non-frail stage. A majority of these respondents reported that frailty was the indicator by which to withdraw certain life-sustaining treatments, however, artificial nutrition and hydration, antibiotics and blood transfusion were not to be stopped under any circumstances. Most of the participants reported that having advance care planning discussion was meaningful and such discussions did not provoke uncomfortable feelings.

In this study, the consistency of decision-making presented a different understanding of patient autonomy in the Taiwanese context. Taiwanese society is heavily influenced by the teaching of Confucius, which emphasizes the importance of maintaining family harmony so that decisions can be made based on the best interests of ‘a family as a whole’ rather than as the interests of ‘an individual’ family member [19,23]. This family-centered approach is often seen in clinical practice and is considered a social norm not only in Taiwan, but also in other Asian countries [6,7,24]. This different interpretation of autonomy challenges the individualistic understanding of autonomy in biomedical ethics derived from Western cultures (i.e., people can make their decisions independently without coercion) [12,25,26]. The argument between the two interpretations is that whether the relational stance of autonomy could be considered a better alternative to reflect the value of collectivism in a non-Western context [27]. A relational autonomy is then proposed and adopted to highlight that an individual’s identities, needs, interests and perspectives are shaped by their relation to others. [11] This may help to explain the findings in this study that patients and family caregivers made similar end-of-life care decisions. Additionally, it cannot be ignored that the patient and family participants in this study had already been aware of the patients’ prognosis and had received palliative care. Therefore, they may already have discussed end-of-life care issues prior to participating in this study and may have had some extent of consensus for care planning, resulting in the possibility of consistent decision-making. More qualitative studies are needed to explore the meaning and underpinning understanding of the decision-making both for patients and family caregivers, which should be a priority for further research. Results of additional research may improve the knowledge of healthcare staff on how to initiate advance care planning discussions and why life-sustaining treatment decisions are made.

Interestingly, almost half of the participants in the present study reported that certain life-sustaining treatments, such as artificial nutrition and hydration, antibiotics or blood transfusion, should not be suspended under any circumstances. This finding appears to be associated with the social norm of ‘filial piety’, which is a primary virtue of taking care of one’s parents or elderly relatives by providing material and emotional support and minimizing physical and psychological distress [23]. In the present study, participants chose to withdraw cardiopulmonary resuscitation and mechanical ventilation because they could possibly be detrimental to patients. However, offering nutrition and fluid was considered to be beneficial from the perspective of family caregivers. Discontinuing artificial nutrition and hydration may be against the family caregivers’ filial responsibility [28]. In addition, the patient or family might not fully understand the actual benefit or harm of administering artificial hydration and nutrition [10]. We suggest that the culturally appropriate adaptation of palliative care should be taken into account by clinicians while initiating advance care planning communication with patients [9]. Furthermore, contextually specific education programs for patients, family caregivers and healthcare staff should be in place to improve the overall awareness and knowledge of palliative care [29,30,31].

Patients and their family caregivers both endorsed early advance care planning discussions, particularly considering the patients’ health condition. Early advance care planning initiative is universal and preferred by patients worldwide. For example, Miyashita et al. [19] reported that more than 70% (n = 365) of Japanese patients visiting outpatient units across four hospitals accepted to have advance care planning discussions prior to illness. The quantitative survey of Kubi et al. [32] which included 200 American cancer patients, demonstrated similar findings that almost half of participants had expressed the willingness to have advance care planning discussions before their cancer diagnosis. Nevertheless, the development of advance care planning in Asia is still at a preliminary stage. Government funding and policy support are urgently needed to enhance the service deployment in routine care so that patients can access the services early on [33].

## 5. Strengths and Limitations

This study has several strengths. First, this study explored preferences regarding advance care planning and life-sustaining treatments decision making by comparing data from two different sources (patients and family caregivers). Second, the questionnaire used in this study was developed and translated based on local cultural context [19]. Third, this study was underpinned by newly rolled-out legislation (Patient Right to Autonomy Act), [14] which facilitated the advance care planning discussion in routine practice to improve its accessibility for patients in Taiwan.

This study has several limitations that require caution when applying the study findings. First, this study was conducted in a single center with a relatively small sample size, which limits the generalization of findings to other populations. However, conducting research with terminally-ill patients is considered challenging, both ethically and practically [34,35,36]. Second, we cannot rule out selection bias as the study participants were those who did not feel reluctant to discuss end-of-life care issues and advance care planning. Third, the patients’ autonomous decisions may be affected by those of family caregivers as they were approached as dyads in the recruitment process. Fourth, the hypothetical scenarios presented in the questionnaire could not address all types of clinical circumstances encountered by patients and family caregivers, which would be challenging for them to imagine and make corresponding decisions. Fifth, the potential discrepancies concerning end-of-life preferences may have been resolved through discussions prior to enrollment into the study as this study was conducted in the palliative care setting and had excluded the refused dyads (*n* = 32) (See Figure 1).

## 6. Implications for Clinical Practice

The study findings can be used to train staff to identify the appropriate timing to facilitate advance care planning discussions and decision-making on life-sustaining treatments for terminally ill cancer patients. Most importantly, the opinions of primary caregivers should be considered while taking care of this patient population in Taiwan.

## 7. Conclusions

Patients’ preferences on the timing of initiating advance care planning and withdrawing life-sustaining treatments are similar and consistent with those of their family caregivers. A majority of participants prefer to have advance care planning earlier when patients are at non-frail disease stage. Patients’ frail disease prognosis indicates the timing for life-sustaining treatment withdrawal except for nutrition and hydration, antibiotics or blood transfusion. The medical decision-making processes in Taiwan appear to be shaped differently by cultural characteristics, including a relational stance toward autonomy and the social norm of filial piety. Qualitative research is needed to further explore these aspects of medical decision making, seeking to understand the meaning behind the decision-making process and should be taken into account while initiating discussions regarding life-sustaining treatments and advance care planning with patients and their family caregivers in the future.

## Figures and Tables

**Table 1:** Demographics and clinical characteristics of patients and family caregivers.

	Patient (*n* = 54)		Family (*n* = 54)	
	***n***	**%**	***n***	**%**
Age, mean (SD)	60.64 ± 13.9		52.57 ± 11.9	
Gender				
Male	30	56	33	61
Cancer diagnosis				
Gastroengerology (Liver, Gallbladder, Peritoneum, Anal, Colon, Gastric, Pancreas)	24	44	-	-
Chest (Lung)	12	22	-	-
Breast	5	9	-	-
Head and Neck (Oral, Nasopharyngeal)	4	7	-	-
Ovary and Endometrial	4	7	-	-
Others	5	9	-	-
Marital status				
Single	11	20	11	20
Married	33	61	39	72
Divorced	6	11	4	7
Bereaved	4	7	0	0
Education years				
Below junior high school	16	30	3	6
Senior high school	12	22	22	41
University	11	20	10	19
Postgraduate	15	28	19	35
Religion				
None	8	15	12	22
Yes	46	85	42	78
Buddhism	28	52	24	44
Christianity	9	17	5	9
Taoism	20	37	22	41
Others	1	2	0	0
Relationship to patients				
Spouse	-	-	18	33
Children	-	-	16	30
Parents	-	-	4	7
Brother/Sister	-	-	11	20
Others	-	-	5	9
Proxy experience				
No	37	69	16	30
Yes	17	31	38	70
Hospital admission experience				
No	24	44	47	87
Yes	30	56	7	13

**Table 2:** Comparison of preferred timing of initiating advance care planning between patients and family caregivers.

	Patient (*n* = 54)		Family (*n* = 54)		
	***n***	**%**	***n***	**%**	***p* Value**
Non-frail period					0.90
too early	1	2	1	2	
a little early	8	15	9	17	
appropriate	43	80	43	80	
a little late	1	2	1	2	
too late	1	2	0	0	
Early pre-frail period					0.90
too early	1	11	1	10	
a little early	5	55	7	70	
appropriate	3	33	2	20	
no need to answer the question	45		44		
Late pre-frail period					0.27
too early	1	17	0	0	
a little early	4	67	3	38	
appropriate	1	17	5	63	
no need to answer the question	48		46		
Frail period					0.35
too early	1	20	0	0	
a little early	2	40	0	0	
appropriate	2	40	3	100	
no need to answer the question	50		51		

**Table 3:** Comparison of the preferred timing for life-sustaining treatments (CPR, intubation and artificial ventilator, artificial nutrition and hydration, antibiotics, blood transfusion and hemodialysis) withdrawal between patients and family caregivers and their perceptions on advance care planning.

	Patient (*n* = 54)		Family (*n* = 54)		
	***n***	**%**	***n***	**%**	***p* Value**
**To stop life-sustaining treatment**					
CPR					0.20
Non frail	13	24	14	26	
Early pre-frail	3	6	3	6	
Late pre-frail	4	7	0	0	
Frail	21	39	21	39	
Don’t stop	4	7	10	19	
Uncertain	9	17	6	11	
Intubation and artificial ventilator					0.19
Non frail	14	26	17	31	
Early pre-frail	5	9	1	2	
Late pre-frail	3	6	0	0	
Frail	21	39	24	44	
Don’t stop	3	6	6	11	
Uncertain	8	15	6	11	
Artificial nutrition and hydration					0.41
Non frail	3	6	6	11	
Early pre-frail	7	13	5	9	
Late pre-frail	1	2	1	2	
Frail	17	31	14	26	
Don’t stop	18	33	25	46	
Uncertain	8	15	3	6	
Antibiotics					0.77
Non frail	3	6	6	11	
Early pre-frail	4	7	5	9	
Late pre-frail	3	6	1	2	
Frail	13	25	10	19	
Don’t stop	27	50	28	52	
Uncertain	4	7	4	7	
Blood transfusion					0.75
Non frail	5	9	5	9	
Early pre-frail	3	6	5	9	
Late pre-frail	4	7	1	2	
Frail	13	24	12	22	
Don’t stop	23	43	26	48	
Uncertain	6	11	5	9	
Haemodialysis					0.73
Non frail	10	19	14	26	
Early pre-frail	5	9	2	4	
Late pre-frail	3	6	2	4	
Frail	16	30	14	26	
Don’t stop	8	15	11	20	
Uncertain	12	22	11	20	
Feel uncomfortable when you discuss ACP with medical personnel at early stage?					0.59
Yes	9	17	9	17	
No idea	3	6	1	2	
No	42	78	44	81	
Discussion ACP is meaningful					0.72
Yes	49	91	51	94	
No idea	4	7	2	4	
No	1	2	1	2	
Attitude change on ACP					N/A
Yes	NA	-	9	17	
No idea	NA	-	5	9	
No	NA	-	40	74	

CPR: cardiopulmonary resuscitation; ACP: advance care planning; N/A: not applicable.

## Appendix A

**Table A1:** Criteria to define different stage of frailty of a cancer patient.

	The Number of Factors of Frailty	The Example Phrases Used in Three Scenarios
Non-Frail	0	“without health problems”
Pre-Frail	1 “Early pre-frail” exhaustion	“be able to walk without cane, t hough he/she got tired more easily than before.”
	2 “Late pre-frail” exhaustion slowness	“got tired much more easily than, and took more time to do daily activities than before.”
Frail	≥3 exhaustion slowness weight loss low physical activity	“physical function was declined and lost the weight of 10 kg. he/she could not get up or stand up without someone’s assists”
